# Flow diversion in challenging vascular anatomies: the use of low profile stent retrievers for safe and accurate positioning of the microcatheter

**DOI:** 10.1186/s42155-020-00106-5

**Published:** 2020-03-30

**Authors:** Ulf Quäschling, Monika Kläver, Cindy Richter, Gordian Hamerla, Simone Mucha, Cordula Scherlach, Jens Maybaum, Karl-Titus Hoffmann, Stefan Schob

**Affiliations:** Department for Neuroradiology, University Hospital Leipzig, Liebigstraße 20, 04103 Leipzig, Germany

**Keywords:** Flow diversion, Aneurysm treatment, Difficult endovascular access, Exchange maneuver, Stent retriever

## Abstract

**Background:**

Hemodynamic therapy with Flow-Diverters has become a fundamental option for treatment of cerebral aneurysms. A major obstacle of Flow-Diverters is the comparatively stiff microcatheter required for implantation. Consequentially, maneuverability is limited and primary catheterization of peripheral targets may be difficult or even futile in challenging vascular anatomies. To overcome this, a highly navigable microcatheter must be used to attain the desired vascular segment, followed by a hardly controllable exchange-maneuver via a long microwire, involving a high risk for wire-perforation. Our study aimed to investigate the value of low-profile stent-retrievers as a railway for introduction of the required microcatheter, which allows to maintain a stable endovascular position and reduce the risk for procedural vessel injury.

**Methods:**

14cases (8females, mean-age 59y) of Flow-Diverter-Implantation requiring the use of a low-profile stent-retriever were reviewed. All cases featured a challenging vascular anatomy. After micro-catheterization of the desired segment, the stent-retriever was carefully deployed as an anchor in a secure, distal location. In all cases a pREset/LITE-stent-retriever was used for introduction of the equipment required for implantation.

**Results:**

In all cases the anchoring-maneuver was performed without technical complications. The stent-retrievers maintained a stable position after deployment in all situations. No potential traumatic sudden movements of the microcatheter occurred. No procedure-related perforations, dissections or vasospasms were observable during the interventions or their aftermath.

**Conclusions:**

In our experience the stent-retriever-anchoring-maneuver represents a potentially essential and safe amendment for flow diverter treatment in technically challenging situations.

## Introduction

Neurovascular stenting using low profile flow diverter stents (FDS) or braided aneurysms stents composed of a less dense mesh for treatment of especially peripheral and small cerebral aneurysms has gained significant importance during recent years (Möhlenbruch et al., 2017; Voigt et al., 2017; Schob et al., 2019a; Martínez-Galdámez et al., 2019; Aydin et al., 2017; Iosif & Biondi, 2019). Extra-aneurysmal flow diversion immediately reduces aneurysmal hemodynamic force by redirecting the physiological blood flow along the natural axis of the parent vessel, away from the aneurysmal orifice. During the further course, a neo-intima is formed along the lattice of the device increasingly embedding its wires into the remodeling vessel wall, eventually causing isolation of the aneurysm from the intracranial circulation.

The uniquely appreciable feature associated with flow diversion (FD) is the peri-procedural avoidance of the highly fragile aneurysm sac. Aneurysm rupture during coiling has been reported to occur only in approximately 4.1% when treating ruptured and in 0.5% when treating unruptured aneurysms, but is associated with a fatal outcome in approximately one third of all cases (Cloft & Kallmes, 2002).

However, microcatheters bearing approval for FD implantation necessarily possess comparatively large inner diameters (0.021″-0.027″) and exhibit considerable catheter stiffness. As a consequence their maneuverability is limited and frequently causes increased mechanical force along the microcatheter during probation of distal vessel segments (Schob et al., 2019a). Therefore, primary probation of the vascular target with the catheter intended for FDS implantation may prove to be very difficult or even futile in demanding vascular anatomies. Hence, microcatheter-exchange maneuvers employing navigation-wise advantageous, small lumen microcatheters (0.0165″) for initial probation are frequently essential prerequisites.

More specifically, a superiorly-navigable, small-lumen microcatheter, for example the Excelsior SL 10 (Stryker Neurovascular, USA) is used to attain the target segment in a first step. Subsequently, as the currently available small lumen is insufficient for delivery of the FD, a long, stiff exchange wire providing enhanced mechanical support is inserted and positioned distally. Then, the most critical step, removal of the smaller microcatheter and introduction of the required microcatheter is performed, trying to maintain an atraumatic distal wire position (Chapot et al., 2013). After the large-bore microcatheter is eventually positioned properly, implantation of the FDS can be performed.

Nevertheless, especially in cases of demanding vascular anatomies, unavoidable movements of the stiff exchange wire due to vessel-straightening, for example accompanied by the advancement of the second, stiffer microcatheter, pose a considerable risk for vessel injury, perforation and loss of a distal wire position already reached. Therefore, a facilitating endovascular strategy is warranted, enabling the endovascular surgeon to safely position the microcatheter for implantation under avoidance of uncontrollable, potentially critical movements of the exchange wire or microcatheter dislocations.

We therefore prospectively collected and retrospectively investigated the safety and feasibility of a low-profile stent-retriever (pREsetLITE, Phenox, Bochum, Germany) as static substitute for otherwise difficult to control, long exchange wires in demanding vascular anatomies.

## Material and methods

### Patients

Our radiological database was reviewed for FDS implantations in the last 2 years, which had been performed including the anchoring employment of a pREset stent retriever. A sum of 213 cases of FDS implantations were performed in this period, however, in only 14 cases (6 male, 8 female patients, mean age of 59 years, ranging from 36 to 69 years) the use of a pREset was eventually necessary. In five cases the anchoring maneuver was performed in the posterior circulation - four times for FD treatment of ruptured dissecting aneurysms of the intradural vertebral artery, one time for treatment of a ruptured basilar artery blister. In nine cases, stent retriever anchoring was performed in the anterior circulation. In two cases the aneurysms were located in the intradural ICA, in five cases at the AcomA complex, in one case at the MCA and in another case at the PcomA origin.

A detailed overview of individual clinical and demographic information is provided in Additional file 1: Table S1.

### Interventional procedure

Oral dual antiplatelet medication (ASA 500 mg and ticagrelor 180 mg) was started the day before the procedure in elective cases. At the day of the procedure the standard regimen, consisting of aspirin 100 mg (once a day, life-long) and ticagrelor 90 mg (twice a day, for 12 months), was inititated.

In case of emergency treatments, intravenous dual platelet inhibition was initiated after interdisciplinary consent was gained for FDS implantation. For this purpose, 500 mg aspirin and 180 μg eptifibatide (Integrilin, GSK) / kg bodyweight were given prior intervention, followed by oral administration of 180 mg ticagrelor after the procedure. The abovementioned standard regimen was continued accordingly.

All endovascular procedures were performed under general anaesthesia using a bi-planar angiography suite. Endovascular access was established via the right femoral artery using an 8-French introducer sheath. A bolus of heparin (5000 IU) was administered via the sheath initially. All procedures were performed by 2 each of 3 available neurointerventionalists with 5, 14 and 18 years of experience.

### The stent-retriever-anchoring maneuver

**Fig. 1:**
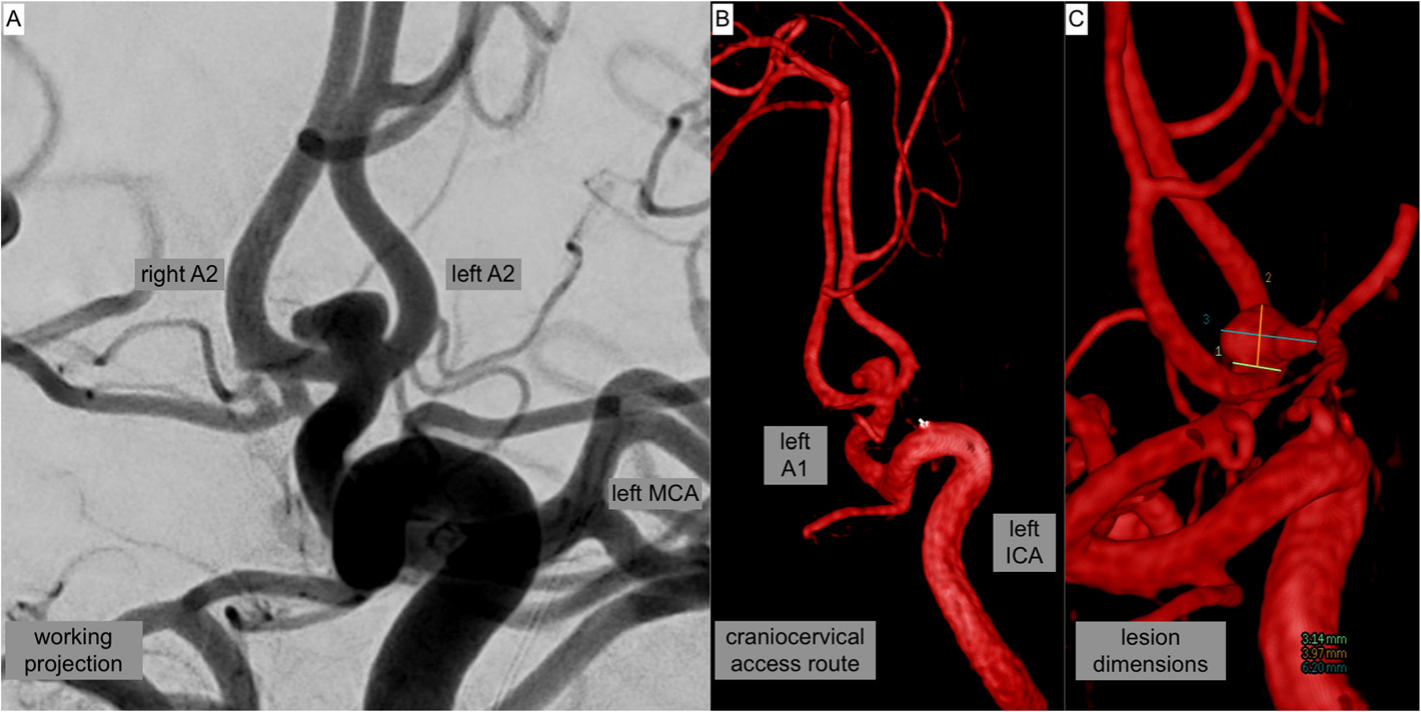
provides DSA images prior treatment. **a** Conventional angiogram in working projection shows a saccular, lobulated, broad based aneurysm of the AcomA-complex (neck: 3.2 mm, fundus: 4 mm × 6.2 mm), predominantly filled through the left handed ICA-ACA. **b** 3D reconstruction of the left ICA demonstrating the cervical and intracranial access route for endovascular treatment**. c** 3D based quantification of the lesion dimensions

**Fig. 2:**
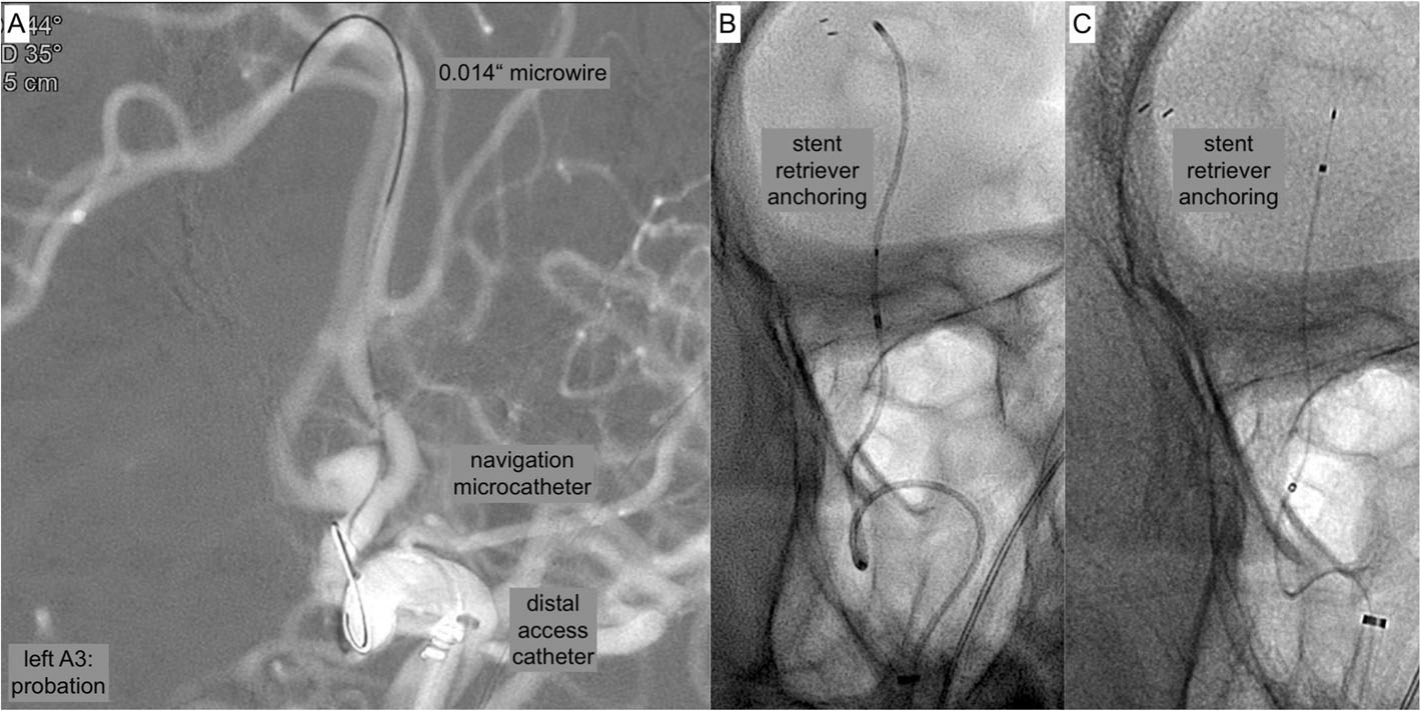
shows representative images of the probation with the 0.0165″ microcatheter and subsequent anchoring of the pREset, preparing the exchange maneuver of the stiffer and less versatile large bore microcatheter required for FDS implantation. **a** Roadmap demonstrating probation of the left handed A3 segment. **b** Plain radiography showing the microcatheter of the initial step and its spatial relation to the vascular anatomy in working projection. **c** Plain radiography showing the microcatheter of the initial step and its spatial relation to the vascular anatomy in a supplementing lateral projection

**Fig. 3:**
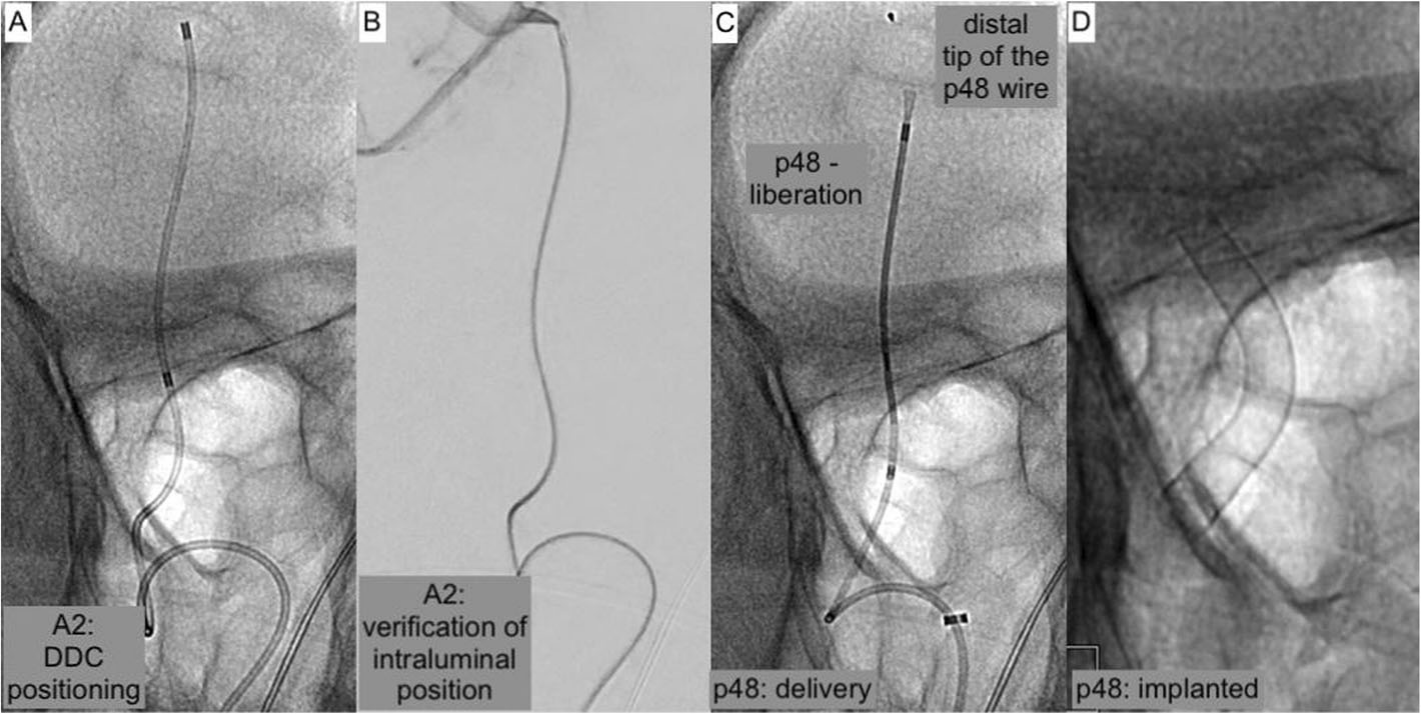
demonstrates the second step of the maneuver – introduction and placement of the greater and potentially more traumatic large bore microcatheter and the succeeding FDS implantation**. a** Plain radiography in working projection shows the placement of the delivery-microcatheter in the distal A2 segment, providing sufficient forerun for the delivery of the densely woven FDS**. b** DSA confirmation of the true lumen by injection of contrast agent via the correctly positioned microcatheter prior implantation**. c** Plain radiography in working projection demonstrating FDS delivery – note the radiopaque olive at the distal end of the p48 wire and the tulip-like appearance of the partially unfolded distal device**. d** Plain radiography of the implanted FDS, optimally covering the aneurysm neck originating from the A1-A2 curve

**Fig. 4:**
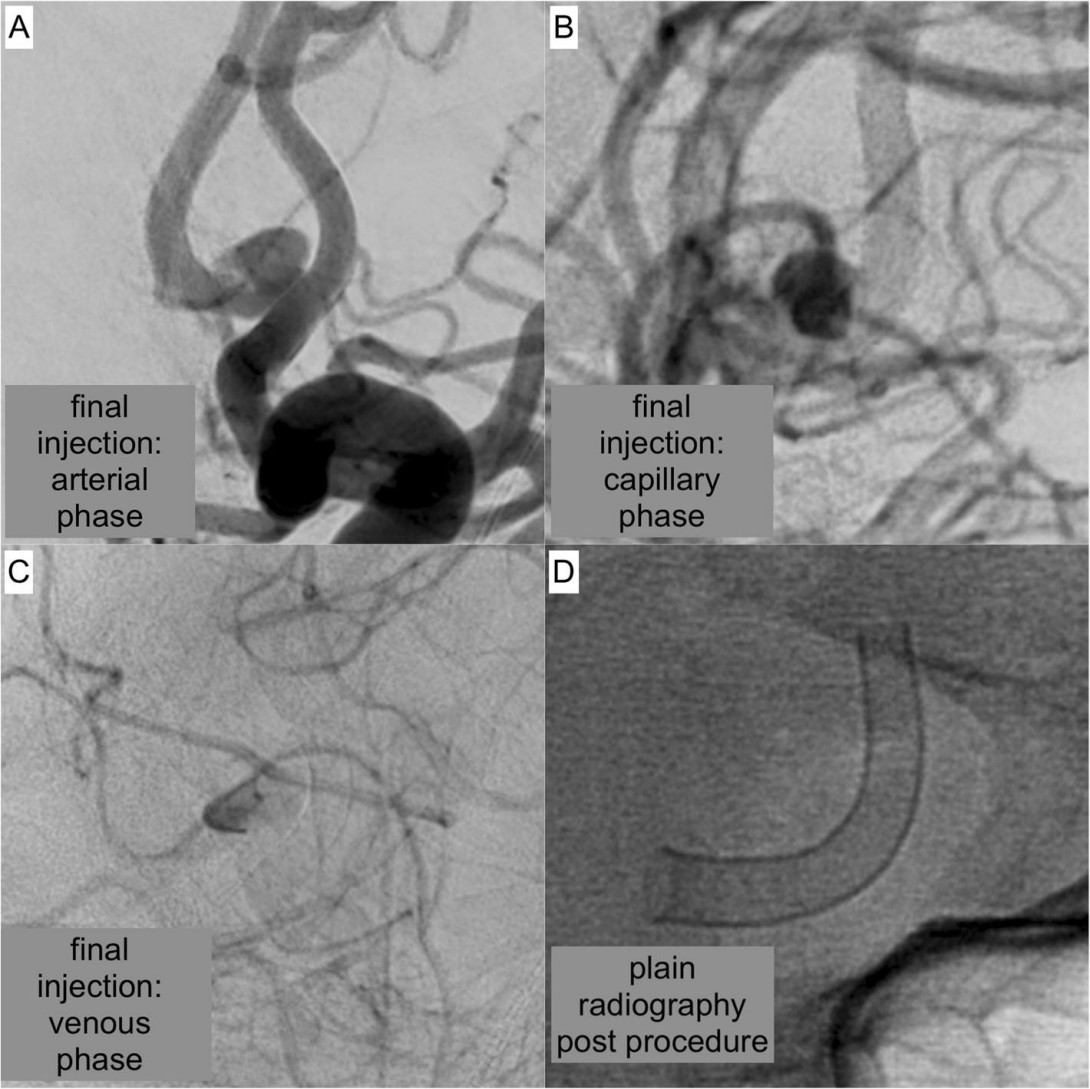
illustrates the result after FDS implantation**. a** DSA image in working projection – arterial phase: the implanted FDS shows ideal wall apposition and optimal coverage of the aneurysm neck. **b** DSA image – capillary phase: extended, strong opacification of the aneurysm. **c** DSA image – venous phase: still persisting filling of the aneurysm sac with contrast agent, indicating high efficacy of the implanted device **d** Plain radiography of the implanted FDS, demonstrating complete and homogeneous unfolding of the device

All cases featured a distinctly curved or otherwise challenging vascular anatomy, which impeded primary catheterization via the microcatheter required for FDS implantation. Except for one case (Prowler Select Plus: Codman Neurovascular, USA), in all remaining patients the Excelsior SL-10 (Stryker Neurovascular, USA) was used for the comparatively demanding catheterization of the target segment in combination with a Traxcess 0.014″ 200 cm microwire (MicroVention Terumo, USA). After reaching the desired segment and confirmation of the correct intraluminal position of the flexible, first microcatheter by contrast injection, the respective pREset / pREset LITE stent-retriever was most carefully deployed for anchoring in a secure, distal location. This way a stable position of the exchange device (pREset / LITE) was achieved and otherwise potentially uncontrollable microcatheter-dislocations or microwire cam-outs were avoided. Figures 1,2, 3, and 4 illustrate the individual steps of the whole maneuver, Additional file 2: Video S1 shows the whole procedure as animation.

Only in one case – for treatment of an extradurally located dissecting aneurysm of the left handed ICA – a conventional pREset (Phenox, Germany) was carefully deployed in the distal ipsilateral M1 segment via the Prowler Select Plus to facilitate the insertion of larger 0.025″ microcatheter (Vasco+ 25, Balt, France), which eventually enabled the smooth implantation of a Silk FDS (Balt, France) for reconstruction of the injured segment.

In all remaining cases the smaller and significantly more versatile pREset LITE was used for microcatheter exchange as described above. Additional file 1: Table S1 provides the individual combination of the microcatheter of all treatments.

The Additional file 2: Video S1 and S2 file illustrates all steps of the anchoring maneuver and FDS implantation in great detail.


**Additional file 2:** Additional video of the preset anchoring maneuver. The video demonstrates each step of the technique in detail.


As an important side note - the introduction and advancement of the second microcatheter along the pREset necessitate distinct preparation, as the push wire is too short for a conventional exchange step. Therefore, after removal of the first microcatheter, the second microcatheter needs to be connected to a 10 ml or 20 ml syringe filled with sterile saline before it is advanced over the short push wire of the stent retriever. This way, the second microcatheter is filled with sterile saline and advancement over the pusherwire – which becomes apparent when it enters the saline-filled body of the syringe – is possible without air embolism. For this specific step a secure anchoring position of the stentretriever, for example in the middle of the M2 segment, is required to prevent dislocation of the device when advancing the microcatheter over the wire without being in control of the pusher wire. Consecutively, as soon as the pusher wire of the pREset emerges from the advanced microcatheter, a flushed hemostatic valve is to be connected to the microcatheter prior pREset removal and subsequent FDS implantation.

## Results

### Patients, aneurysm location and microcatheter

Four of the cases were treated for acute aneurysmal SAH secondary to a ruptured dissecting aneurysm of the intra-dural vertebral artery using FDS-implantation as reconstructive strategy (Schob et al., 2019b). One case received FDS-treatment for reconstruction of a non-ruptured, but hemo-dynamically significant dissecting aneurysm of the left internal carotid artery in the petrous segment. Five patients were treated electively for innocent aneurysms of the AcomA complex. One patient was treated for an innocent MCA-bifurcation aneurysm and a further one for an innocent ICA aneurysm in the cavernous segment. Finally, two patients were treated for incidental aneurysms in the posterior circulation (1x PcomA, 1 x basilar artery).

### Implanted devices

Of those 14 patients the majority received a p48 MW (Phenox, Germany, *n* = 7) for aneurysm treatment. Two patients were treated with a Pipeline Embolization Device 2 FDS (PED2, Medtronic, USA), two had a p64 FDS (Phenox, Germany) and three a conventional Silk (Balt, France) implanted.

### Microcatheters used for primary catheterization

#### And stent retrievers employed as anchoring devices

In all but one (Prowler Select Plus for the above mentioned conventional pREset) cases the Excelsior SL-10 was used for the distinctly challenging catheterization of the target segment in combination with a Traxcess 0.014″ microwire. After reaching the desired segment, the pREset stent retriever (13x pReset LITE, 1 x pREset) was atraumatically deployed for anchoring. This way a stable position of the exchange device was achieved and insertion of the significantly stiffer and less versatile second microcatheter was facilitated.

#### Microcatheters used for implantation

For implantation of all p48 MW FDS the Prowler Select Plus microcatheter was employed. For implantation of all PED2 FDS the Phenom™27 (Cathera, USA) was used. For p64 implantation, one Excelsior®XT-27 (Stryker Neurovascular, USA) and one Phenom 27 were used. Implantation of conventional Silks was performed with the Vasco+ 25 in two cases and with the Vasco+ 21 (Balt, France).

### Technical complications

In all cases the *pREset anchoring maneuver* was performed without encountering significant technical pitfalls. All pREsets were smoothly deliverable and maintained a stable position after deployment in all situations. Even the cases requiring positioning of especially firm 2.7F microcatheters (Phenom 27, Excelsior XT27), no potentially traumatic sudden movements of the microcatheter or stent retriever occurred. As a consequence, no procedure related perforations, perceptible dissections as well as device-induced vasospasms were observable during the interventions or their aftermath.

### Outcome and follow ups

Our routine-follow up regimen after FDS implantation warrants a native cranial CT 24–48 h after implantation in clinically unconspicuous patients. In case of delayed regaining of consciousness post general anaesthesia, novel focal neurological deficits or behavioural abnormalities, cranial MRI including diffusion weighted imaging, T2weighted imaging and T2*weighted- or susceptibility weighted imaging is amended. Further regular DSA imaging is performed 3–9 months and 24 months after implantation, depending on the implanted device and the patient’s preference.

In our patient cohort, one patient experienced a transient, subtle lower limb paresis after endovascular treatment of an aneurysm at the A1-A2 junction, which had resolved completely 3 days post intervention. Cranial MRI revealed few DWI and FLAIR positive spots in the peripheral ACA territory, representing small thromboembolic-ischemic events. Otherwise, no further patient experienced any procedure-related complications. Most importantly, no worsening of post procedure mRS or morphological evidence of procedural-iatrogenic vascular injury was observable.

## Discussion

FD has become a highly efficient standard treatment for most intracranial sidewall aneurysms (Dmytriw et al., 2019). However, the delivery of the extra-saccular endovascular implants to sometimes quite peripherally located vascular targets may prove particularly challenging, especially when encountering significant proximal supra-aortic elongation along with unfavorable distal junction angles at the transition from the proximal to the distal segments of the cerebral vessels (Schob et al., 2019a; Martínez-Galdámez et al., 2019). In those cases, exchange maneuvers are required to eventually provide a sufficiently large lumen for device delivery in combination with stable access to the target segment (Chapot et al., 2013). Conventionally, those exchange maneuvers are performed under greatest precaution using a long 300 cm measuring exchange wire, which allows – to a limited extent – controlled maintenance of the distal endovascular position whilst extra-corporeally substituting the first, smaller microcatheter for the second, larger microcatheter. The microcatheter substitution along the exchange wire involves a significant risk for vascular injury, distal wire perforation and subsequent SAH (Chapot et al., 2013). An additional, certainly less critical but rather frequently encountered complication is the loss of distal endovascular access during the exchange maneuver, as the propulsive motion of the second microcatheter eventually causes unintended retraction of the distally placed wire, or the microwire simply does not provide adequate mechanical support to advance the large bore microcatheter to its desired final position.

Aiming to propose a solution for this problem, our work provides a precise protocol of our stent-retriever anchoring maneuver along with the evaluation of its technical and clinical outcomes of accordingly performed cases in our center.

Most importantly, we did not experience any significant technical or clinical complications related to the stent retriever augmented FDS implantation in any of the cases. More precisely, we did not observe any unintended, potentially harmful dislocations of the stent retriever during the procedure. Furthermore, each pREset provided a reliably stable monorail delivery platform for precise and atraumatic microcatheter positioning.

However, comparable to other stent retrievers, the push wire of the pREset system only provides a useable length of approximately 180 cm for microcatheter exchange, compared to 300 cm of the specifically designed exchange wires. This issue requires particular attention of the interventionalist, as the advancement of the second microcatheter must be performed without being in control of the proximal, extra-corporeal end of the exchange platform. In our experience this handicap is compensated by the reliably stable endovascular position of the adequately sized stent retriever shaft, which prevented hazardous dislocations bearing the risk for vessel wall injury or even traumatic SAH in all of our cases.

To our best knowledge, this is the first systematical analysis of the use of stent retrievers as stable and superiorly controllable exchange-platform for technically challenging cases of flow diverter implantation. However, other facilitative, distal anchoring techniques – including coil-anchoring and balloon-anchoring etc. – mostly for endovascular treatment of giant aneurysms, have been reported earlier (Effendi et al., 2016; Oran et al., 2013; Fargen et al., 2013; Snyder et al., 2010). Also, the use of stent retrievers for advancement of large-bore aspiration catheters through tortuous, proximal vessels in the context of intracranial revascularization has been demonstrated previously (Lin et al., 2019; Singh et al., 2015; Mascitelli et al., 2016) and is continuously applied successfully in the clinical routine. Especially the fortifying experience with low profile stent retrievers in stroke treatment (Kurre et al., 2017), more precisely their outstanding distal stability and low risk profile, inspired us to implement them as exchange platforms in technically otherwise hardly controllable scenarios.

Our study suffers from a number of limitations – most importantly it is only based on a single center experience with retrospective design, thus offering intrinsically narrow information on the performance of this approach, which was gained with a comparatively small number of cases. Therefore, further studies involving additional experienced centers are wanted to corroborate our findings.

## Conclusion

In our experience the low-profile stent-retriever anchoring maneuver represents a potentially essential and safe amendment for FDS-implantation in technically especially challenging situations. A docking wire for low profile stent retrievers like the pREset LITE - to enhance the length of the system for improved exchange maneuvers – would significantly facilitate the technique.

## Additional files

**Additional file 1:Table S1.** Detailed overview of technical and clinical aspects of included cases.

## Data Availability

All relevant data is provided in the manuscript. However, additional supplementary data can be provided from the corresponding author upon request.
