# Supplementary material for: Flow diversion in challenging vascular anatomies: the use of low profile stent retrievers for safe and accurate positioning of the microcatheter
Source: CVIR Endovasc. 2020 Mar 30;3:19. doi: 10.1186/s42155-020-00106-5 (PMC7103572; doi:10.1186/s42155-020-00106-5)
Supplement: Supplementary file 2 — Additional file 1:Table S1. Detailed overview of technical and clinical aspects of included cases. [file 42155_2020_106_MOESM1_ESM.docx]

| **Case** | **Lesion** | **Microcatheter employed for**  **initial probation** | **Device employed for exchange** | **Microcatheter**  **employed for device delivery** | **final endovascular**  **implant** | **24h imaging findings** | **clinical follow up – time point** | **Change in mRS related to procedure, final mRS** |
| --- | --- | --- | --- | --- | --- | --- | --- | --- |
| 1 | ruptured dissecting aneurysm: left V4, dominant segment | Excelsior SL 10 | pReset lite 4-20 | Phenom 27 | p64 | unremarkable | 4 months | none, 2 |
| 2 | ruptured dissecting aneurysm:  left V4 dominant segment | Excelsior SL 10 | pReset lite 4-20 | Excelsior XT-27 | p64 | unremarkable | 5 months | none, mRS 0 |
| 3 | ruptured dissecting aneurysm: left dominant V4 | Excelsior SL 10 | pReset lite 3-20 | Phenom 27 | PED2 Flex | unremarkable | 4 months | none, mRS1 |
| 4 | ruptured dissecting aneurysm: left dominant V4 | Excelsior SL 10 | pReset lite 3-20 | Phenom 27 | PED2 Flex | unremarkable | 6  months | none, mRS0 |
| 5 | dissecting aneurysm: left ICA, petrosal segment | Prowler select plus | pReset 4-20  4-20 | Vasco 25 | SILK | unremarkable | 6 | none, mRS0 |
| 6 | ruptured saccular aneurysm: left A1-A2 junction | Excelsior SL-10 | pReset lite 3-20 | Vasco 21 | Silk mini | unremarkable | 6 | none, mRS0 |
| 7 | incidental saccular aneurysm: right ICA, cavernosal segment | Excelsior SL-10 | pReset lite 4-20 | Vasco 25 | Silk | unremarkable | 6 | none, mRS0 |
| 8 | incidental saccular aneurysm: left A1-A2 junction | Excelsior SL-10 | pReset lite 3-20 | Prowler select plus | p48 MW | few distal thrombo-embolic infarctions | 9 | transient leg paresis, mRS0 after 10days |
| 9 | incidental saccular aneurysm: left A1-A2 junction | Excelsior SL-10 | pReset lite 3-20 | Prowler select plus | p48 MW | unremarkable | 1 | none, mRS0 |
| 10 | incidental saccular aneurysm: left MCA bifurcation | Excelsior SL-10 | pReset lite 3-20 | Prowler select plus | p48 MW HPC | unremarkable | 1 | none, mRS0 |
| 11 | incidental saccular aneurysm: distal basilar artery | Excelsior SL-10 | pReset lite 3-20 | Prowler select plus,  Scepter C compliant ballon | p48 MW HPC | unremarkable | 1 | none, mRS0 |
| 12 | incidental saccular aneurysm:  left A1-A2 junction | Excelsior SL-10 | pReset lite 3-20 | Prowler select plus | p48 MW HPC | unremarkable | 1 | none, mRS0 |
| 13 | incidental saccular aneurysm:  left A1-A2 junction | Excelsior SL-10 | pReset lite 3-20 | Prowler select plus | p48 MW HPC | unremarkable | 1 | none, mRS0 |
| 14 | incidental saccular aneurysm:  right PcomA | Excelsior SL-10 | pReset lite 3-20 | Prowler select plus | p48 MW HPC | unremarkable | 1 | none, mRS0 |
